# User-defined morphogen patterning for directing human cell fate stratification

**DOI:** 10.1038/s41598-019-42874-8

**Published:** 2019-04-23

**Authors:** Mary C. Regier, Jacob J. Tokar, Jay W. Warrick, Lil Pabon, Erwin Berthier, David J. Beebe, Kelly R. Stevens

**Affiliations:** 1 Department of Bioengineering, University of Washington, 98195 Seattle, USA; 2 Institute for Stem Cell and Regenerative Medicine, University of Washington, 98109 Seattle, USA; 3 Department of Biomedical Engineering, University of Wisconsin - Madison, 53706 Madison, USA; 4 Carbone Cancer Center, University of Wisconsin - Madison, 53792 Madison, USA; 5 McArdle Laboratory for Cancer Research, University of Wisconsin - Madison, 53705 Madison, USA; 6 Department of Pathology, University of Washington, 98195 Seattle, USA; 7 Department of Chemistry, University of Washington, 98195 Seattle, USA

**Keywords:** Stem-cell biotechnology, Assay systems, Pattern formation, Pluripotent stem cells

## Abstract

Concentration gradients of biochemical stimuli such as morphogens play a critical role in directing cell fate patterning across species and throughout development but are not commonly recapitulated *in vitro*. While *in vitro* biomolecule gradients have been generated using customized microfluidic platforms, broad implementation has been limited because these platforms introduce new variables to cell culture such as externally driven flow, culture in a specialized matrix, or extended time for *in situ* long range diffusion. Here we introduce a method that enables preforming and then transferring user-controlled gradients to cells in standard “open” cultures. Our gradient patterning devices are modular and decoupled from the culture substrate. We find that gradient generation and transfer are predictable by finite element modeling and that device and loading parameters can be used to tune the stimulus pattern. Furthermore, we demonstrate use of these devices to spatially define morphogen signal gradients and direct peri-gastrulation fate stratification of human pluripotent stem cells. This method for extrinsic application of biochemical signal gradients can thus be used to spatially influence cellular fate decisions in a user-controlled manner.

## Introduction

Spatial and temporal patterns of biochemical signals play a central role in orchestrating the development of multicellular organisms. Such signals, termed “morphogens”^1^, have been shown to act through dynamic concentration gradients to drive embryonic fate specification and patterning in model organisms^2–4^. For example, during the gastrulation phase of embryonic development, morphogen gradients acting through transforming growth factor-beta (TGF-β, e.g. Activin/Nodal and bone morphogenetic protein 4, BMP4), Wnt, and other signaling pathways direct germ layer specification and organize developmental body axes^5–7^.

Insights gained in model organisms have been applied to direct fate specification of *in vitro* cell populations, such as human pluripotent stem cells (hPSCs)^8^. In such studies, small molecules or macromolecules that activate or inhibit developmental pathways (e.g., TGF-β and Wnt signaling) are often administered to hPSCs by addition to cell culture media^9–11^. When these media are applied in “macroscale” open cell cultures, turbulent mixing and convective currents in the overlaid media^12^ disrupt prior patterning of dissolved factors. As a result, most hPSC directed differentiation methods include the choice, concentration, and timing of biochemical stimulation, but they do not allow the user to determine spatial patterning of soluble signals within individual cell culture wells^13,14^.

To induce spatial fate stratification in hPSC cultures, several groups have shown that geometric confinement of hPSC colonies *in vitro* induces fate organization along the culture radius^15–19^. For example, when treated uniformly with morphogens such as BMP4, these cultures exhibit concentric zones of expression for ectoderm, mesendoderm, and extraembryonic fate markers in a manner that mimics fate ordering in a gastrulating embryo. This patterning is thought to arise through cell-driven patterning of morphogen (BMP4) and antagonist (Noggin, BMP antagonist) gradients across confined colonies^18,20,21^. Further, varying the timing or concentration of BMP4, Wnt, and Activin/Nodal morphogens or the size, density, or shape of the colony can elicit varying radial distribution of downstream signals and subsequent differentiation patterns across the hPSC colonies^15–24^. While these studies provide informative *in vitro* models of self-driven peri-gastrulation fate patterning, they rely upon cell-directed signal patterning that occurs after homogenous application of soluble stimuli to the medium. Thus, these studies have not allowed the user to directly define the spatial presentation of morphogens to stratify peri-gastrulation cell fates.

In order to more directly achieve spatial and temporal control over morphogen gradients, a number of groups have used microscale culture approaches. For example, patterned stem cell differentiation has been performed in flow-based microfluidic gradient generators^25–28^. Although these systems enable gradient formation, fluid flow disrupts secondary, cell-derived signal patterns^28^ and exposes cells to fluid shear^29^, both of which influence differentiation. Other groups have avoided issues associated with flow by patterning differentiation using morphogen gradients generated through source-to-sink diffusion in hydrogels^30–32^. In these systems, cells are exposed to new matrices as well as to the morphogen itself while the gradient forms and stabilizes within the matrix (a time period that varies based on the biochemical cue’s molecular weight and matrix porosity). Thus, while these technologies have taken important steps forward towards creating user-defined gradients, they typically introduce new variables into hPSC cultures.

We sought to build on this previous work by creating an accessible method to directly control cell lineage stratification *in vitro* by generating and then rapidly transferring tunable morphogen gradients to hPSCs in open culture. Our method includes tunable parameters such as device geometry and dosing regimen that enable the user to directly control the shape, magnitude, and stability of applied morphogen gradients. Importantly, our approach decouples the patterning matrix of a passive diffusion-based gradient generator from the cell culture substrate. Such decoupling enables the use of substrate conditions (i.e., Matrigel coated substrates) and upstream and downstream manipulations and endpoints (i.e., culture fixation and staining, continued culture, or dissociation and recovery) commonly used in protocols for directing and analyzing hPSC fate specification. We use this method to demonstrate that extrinsic morphogen gradient stimulation spatially orders early hPSCs fate decisions in a user-defined manner.

## Results

### Design and fabrication of gradient patterning devices

**Figure 1:**
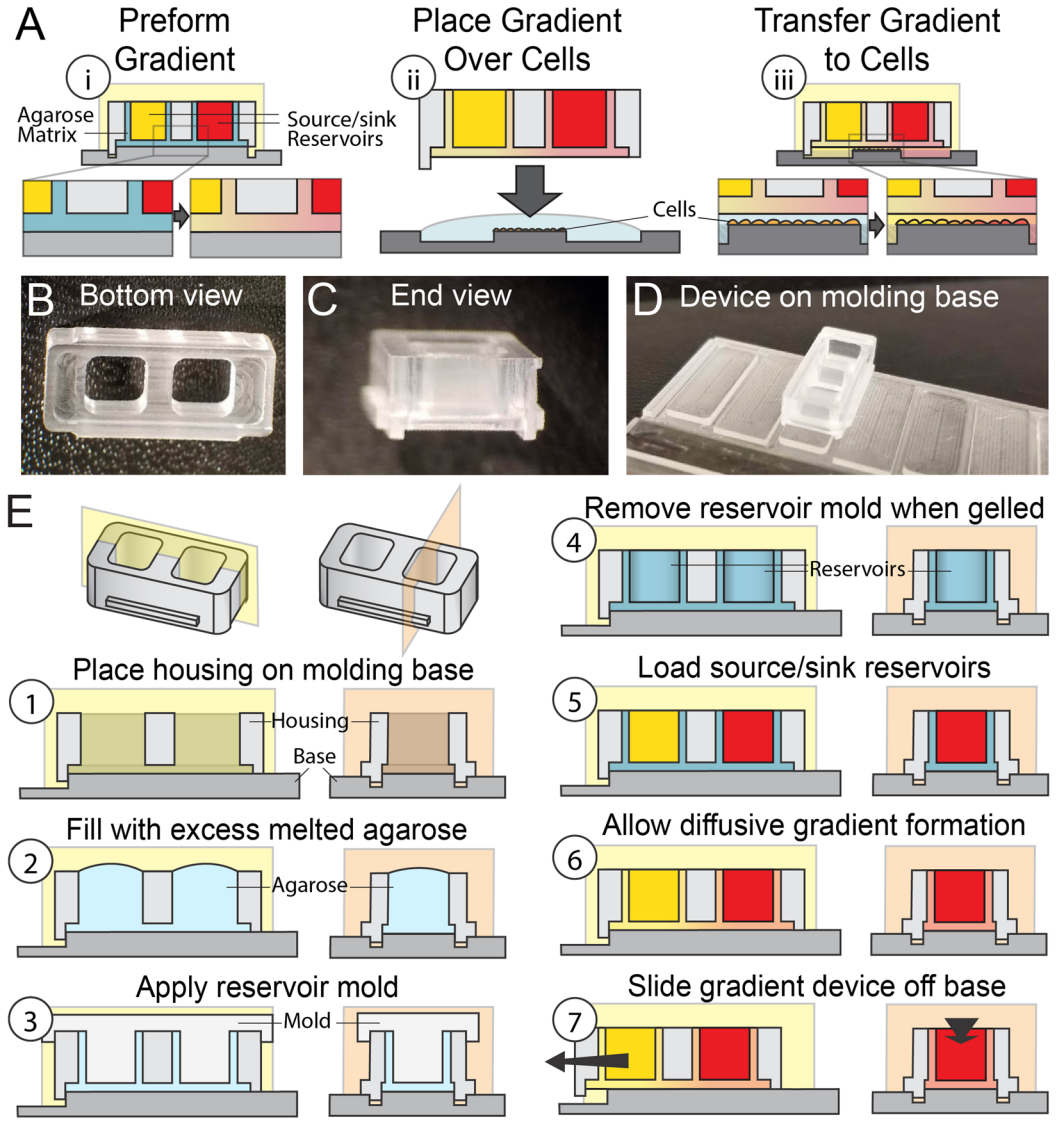
Approach and devices for gradient formation and transfer to cells. (**A**i) Micromachined gradient device-contained source and sink reservoirs were lined with agarose (blue) and connected by an agarose sheet (blue). (**A**ii) Gradients were formed in the agarose sheet of the device via diffusion between the device’s loaded reservoirs. (**A**iii) Gradient-containing devices were placed above cells, and biomolecules were transferred from the agarose sheet to cells by diffusion. Photographs of polystyrene housings showing device (**B**) bottom view, (**C**) end view, and (**D**) assembly with molding base. Diagrams in (**E**) depict the steps for molding the agarose gel (E, steps 1–4), forming the gradient (E, steps 5 and 6), and removing the device for placement over cells (E, step 7).

We developed a system to prepattern transferable biomolecule gradients within agarose matrices that could remain physically separated from cultured cells and their substrates. Our approach started with offline gradient preformation in a molded agarose hydrogel (Fig. 1Ai, blue) between source and sink reservoirs (Fig. 1Ai, yellow and red compartments). The gradient-containing hydrogel device could then be removed from the molding base and placed over cells on a substrate (Fig. 1Aii). A thin layer of media (100 µm height) separated underlying cells from the gradient-containing agarose gel, which enabled pattern transfer from the device to cells by diffusion (Fig. 1Aiii).

To do this, we used micromilling to fabricate polystyrene (PS) housings (Fig. 1B,C) and molding bases (Fig. 1D) as well as polymer casting (polydimethylsiloxane, PDMS) to fabricate reservoir molds. Using these components, we fabricated devices which included two molded agarose gel cups addressable from the top side and connected by a thin sheet of gel on the bottom side of devices. Briefly, PS housings were placed on molding bases used to define the bottom surface of the agarose hydrogel (Fig. 1E step 1). The placement of referencing feet on the side of the housing and the relative height of the bottom molding surface allowed for precise positioning of the agarose gradient matrix in the Z-direction. To form the agarose structure within the housing excess melted agarose (light blue) was added to the voids of the housing (Fig. 1E step 2) and a PDMS reservoir mold (light gray) was placed into the reservoir voids of the housing before gelation occurred (Fig. 1E step 3). The reservoir mold thereby defined the walls of the source and sink cups (Fig. 1E step 3). Once the agarose matrix (blue) had cooled and gelled the reservoir mold was removed to expose empty source and sink reservoirs in the agarose matrix (Fig. 1E step 4), which could then be filled with source and sink solutions (Fig. 1E step 5, yellow and red compartments). In these devices, one cup was filled with biomolecule (the source), which diffused through the hydrogel sheet towards the second cup (the sink). This source-sink configuration resulted in the formation of a gradient in the gel sheet between the cups (Fig. 1E step 6). The relative difference in source/sink reservoir volume compared to the gradient-forming gel sheet volume allowed for gradient stabilization. Following gradient formation, the device was removed from the molding base (Fig. 1E step 7) and inserted above cells in culture (Fig. 1Aii). The high internal fluidic resistance of the gradient-containing agarose hydrogel^33^ stabilized the preformed gradient during device removal from the molding base and placement above the cell culture substrate. Reliefs around culture surface islands received the gel housings, and referencing feet facilitated positioning of the bottom transfer surface of the gel 100 µm above the culture surface. The gradient pattern was then transferred by diffusion from the gel to the cells over the desired culture period (Fig. 1Aiii). Following gradient transfer, the device was removed, making treated cell cultures easily accessible for manipulation according to standard practices.

### Modeling, validating, and tuning biomolecular gradients

**Figure 2:**
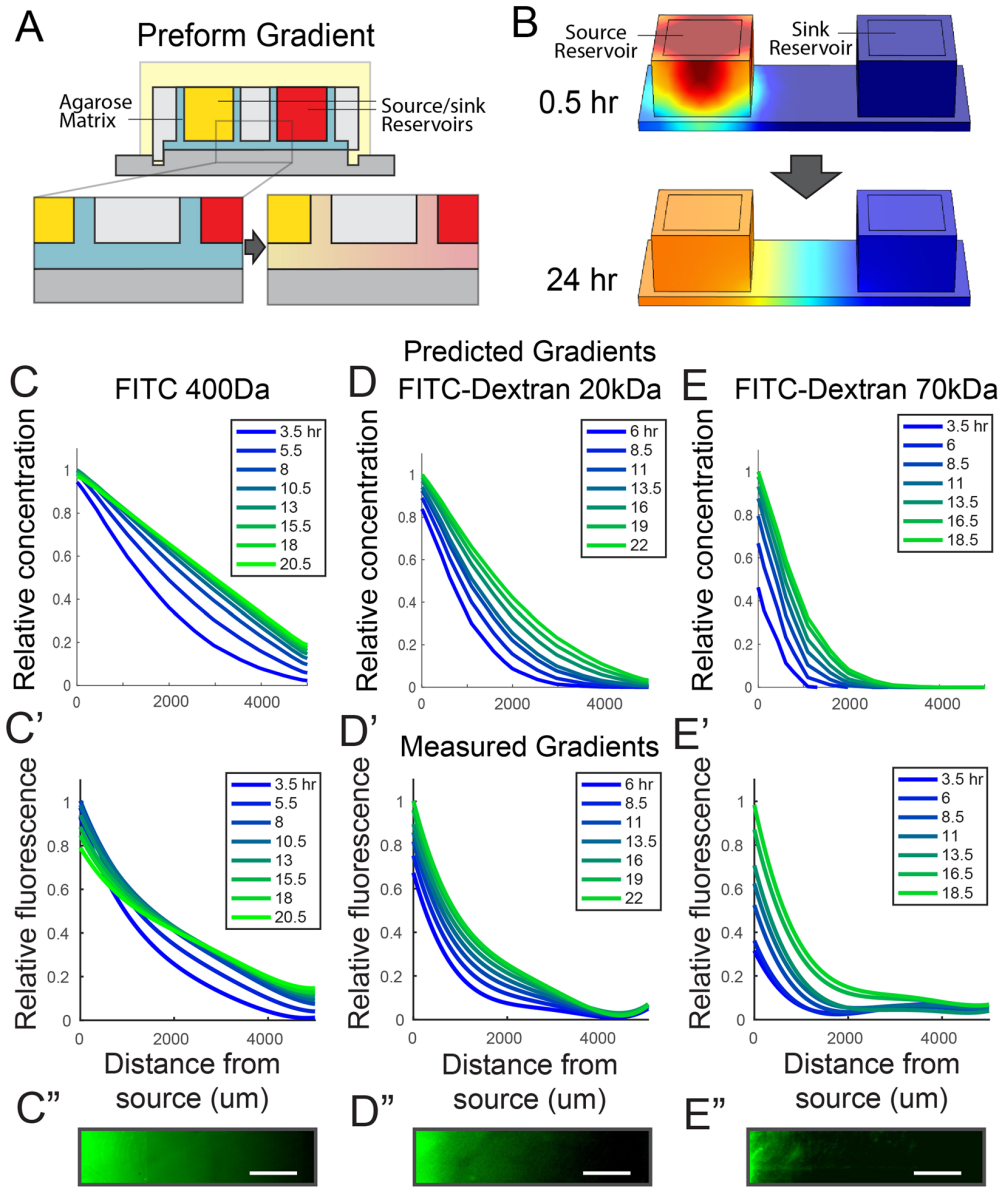
Prediction and verification of gradient formation dynamics. (**A**) The stage of offline gradient preformation was (**B**) modeled in Comsol to predict gradient profile progression for fluorescent or fluorescently tagged molecules (**C**) FITC, (**D**) FITC-Dextran 20 kDa, and (**E**) FITC-Dextran 70 kDa using published diffusion properties for 3% agarose, FITC, and dextrans. The corresponding empirical measurements of gradient formation made using fluorescence microscopy for (**C’**) FITC, (**D’**) FITC-Dextran 20 kDa, and (**E’**) FITC-Dextran 70 kDa were in agreement with modeled dynamics. Representative micrographs across the gradient forming region are displayed in (**C”**–**E”**). Scale bars: (**C”**–**E”**) 1 mm.

We first set out to predict and characterize the formation of gradients within our patterning devices prior to the transfer of gradients to cells (Fig. 1Ai). Based on other studies in which hydrogel matrices were used as media for gradient formation, we reasoned that gradient shape in our devices would be influenced by properties of the diffusing biomolecules and modifications to the device design (e.g., distance between source and sink)^33–36^. To investigate the influence of these parameters we implemented finite element modeling (FEM) including our device geometry and published diffusion properties of the hydrogel matrix and comparable solutes (Fig. 2A,B)^37–40^. We then tested whether gradient generation proceeded as predicted by FEM. Indeed, for the formation of gradients of detectable fluorescently tagged molecules of varying sizes (400 Da to 70 kDa) within the device, modeling results (Fig. 2C–E) agreed with corresponding empirical data (Fig. 2C’–E”).

**Figure 3:**
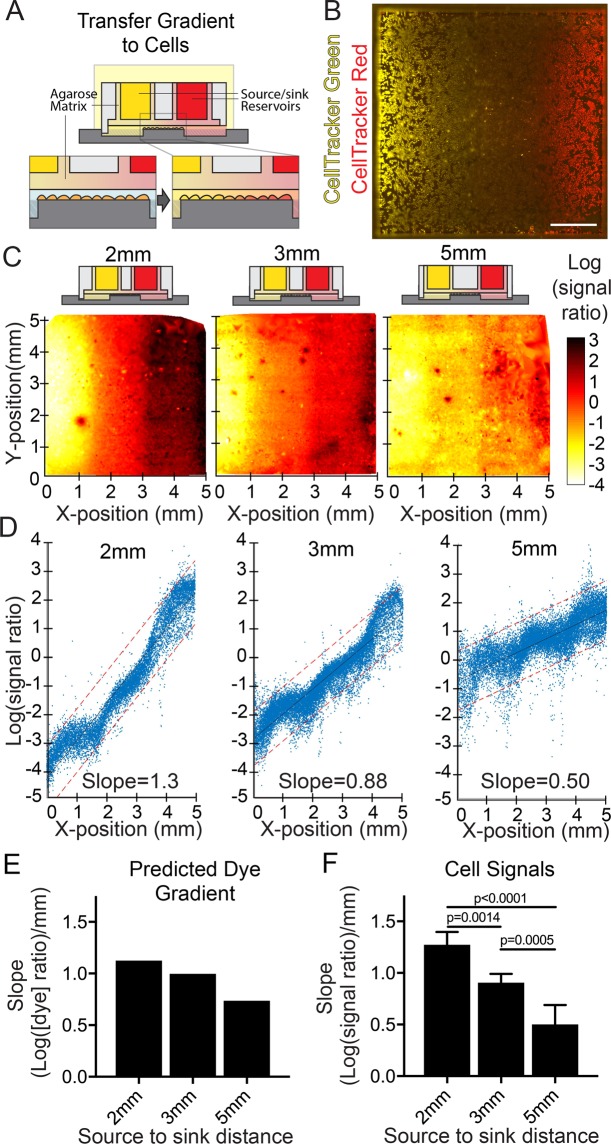
Tunable gradients can be transferred to cells. (**A**) The stage of gradient transfer from the agarose sheet to cells by diffusion and gradient tuning through device geometry was characterized using (**B**) CellTracker dye gradients transferred to human umbilical vein endothelial cells (HUVECs; Yellow, CellTracker Green; Red, CellTracker Red). (**C**,**D**) The slope of the gradient transferred to HUVECs could be tuned by modifying the separation distances between gradient device source reservoirs (2, 3, or 5 mm separation, respectively; single cell log(signal ratio) quantification mapped to scale ranging from −4 – white to 3 – black). (**E**) The trend in slope change predicted by FEM was matched by (**F**) experimental in-cell signal quantification (mean ± SEM for N = 6). Scale bar: (**B**) 1 mm.

We next sought to apply our FEM framework to predict CellTracker label patterning in cell monolayers after the diffusive transfer of gradients (tuned by modifying device geometries) to cell monolayers (Figs 1Aii,iii and 3A,B). Following application of patterned gradients over monolayer cultures of human umbilical vein endothelial cells (HUVECs), we noted variability in cells’ CellTracker signals across a given X-position and even in adjacent cells, which we attributed to cell-to-cell variability in uptake and conversion of the dyes (Fig. S1). To better analyze and control for cell-to-cell differences in labeling efficiency and thus better assess gradient exposure across the population, we applied opposing gradients of two different CellTracker dyes from the same device. This strategy enabled us to quantify the proportion of two fluorescent signals in individual cells corresponding to patterned dye concentration ratios and thereby normalize for varied CellTracker retention efficiencies between cells. We characterized gradient transfer with the log() of the ratio of the resultant signals from both dyes in each individual cell (rather than absolute signals from just one dye, Figs 3B, S1). We reasoned that division of an increasing signal curve by an opposing decreasing signal curve would result in an exponential relationship between the signal ratio and X-position. As predicted, for transfer of opposing cell dye gradients, the log() of the CellTracker signal ratio for individual cells (Fig. 3C) *versus* each cell’s X-position could be fit with a line (Figs 3D, S1). Furthermore, the slope of the line could be tuned by changing the distance between the reservoirs, with slope decreasing (gradients becoming less steep) as the reservoir separation increased (Fig. 3D). Finally, the calculated slopes for gradients transferred to cells using devices with varying reservoir separations followed FEM predictions (Fig. 3E,F). Together, these data demonstrate our ability to predictably generate gradients with user-defined slope and to transfer these gradients to cells in culture.

### Exogenous morphogen gradients trigger spatial ordering of hPSC fates

**Figure 4:**
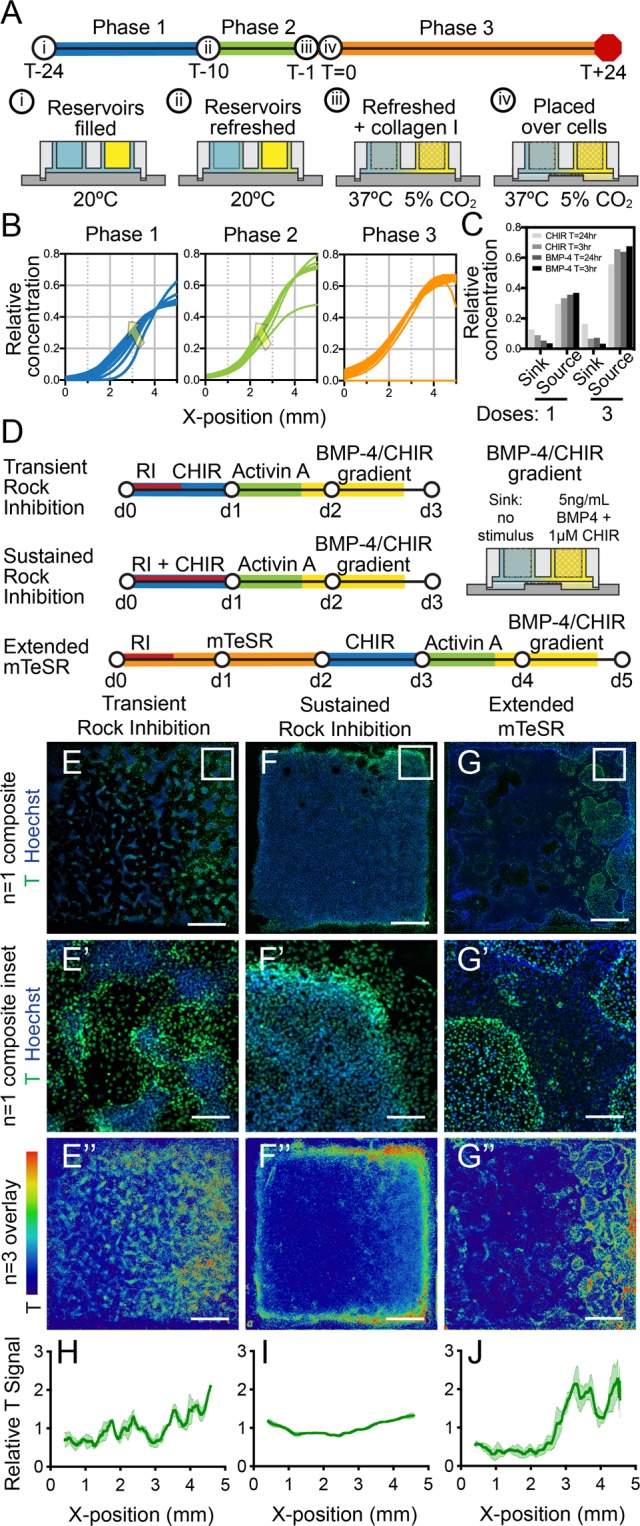
External morphogen gradients drive hPSC fate patterning in a manner that integrates with initial culture conditions. (**A**) A multiple dosing approach was used to form a stable and robust gradient of both small molecules and macromolecules (e.g., protein). (**B**) FEM predicted the formation and progressive stabilization of a BMP4 gradient over the first two diffusion phases (arrows denote direction of gradient profile change over time) and a stable gradient during the third phase (gradient transfer to cells). (**C**) Modeling of CHIR99021 and BMP4 diffusion dynamics demonstrated an increase in source to sink concentration difference for the multiple dosing (three doses) method versus a single dose. (**D**) Human pluripotent stem cells (hPSCs) were cultured under three different upstream culture regimens prior to exposure to a BMP4/CHIR99021 gradient (RI, Rock inhibitor Y-27632; CHIR, CHIR99021). (**E**–**G”**) All conditions generated global Brachyury expression (T, green) profiles in which T was higher at the source and lower at the sink end of the gradient, respectively. However, finer features of expression patterns varied with upstream culture method. (**E**–**E”**) transient rock inhibition, diffuse gradient; (**F**–**F”**), sustained rock inhibition, gradient with strong edge effect; and (**G**–**G”**) extended mTeSR culture conditions, irregular gradient. (**E**–**G**) are representative N = 1 images across the full 5 mm × 5 mm wells, (**E’**–**G’**) are higher magnification images of the regions designated in (**E**–**G**), and (**E”**–**G”**) are overlays of Brachyury signals across N = 3 islands visualized with ImageJ’s physics LUT scale. (**H**–**J**) Brachyury signals relative to Hoechst signals were quantified for the N = 3 replicate cultures overlayed in (**E”**–**G”**) and plotted as the mean with SEM represented with shading. Scale bars: (**E**–**G**) and (**E”**–**G”**) 1 mm; (**E’**–**G’**) 150 μm.

To use this method of gradient formation and transfer to achieve spatial control over cell fate decisions, we next patterned biochemical modulators of hPSC lineage specification *in vitro*. Towards this end, we sought to create gradients of the growth factor, BMP4 (40 kDa), and the GSK3 inhibitor and Wnt agonist, CHIR90021 (≈400 Da) – biomolecules which are commonly used to induce early cell fate specification in hPSCs^41–43^. We adapted our approach to co-pattern these macro- and small molecules simultaneously despite their differences in diffusivity. We hypothesized that a multiple dosing regimen, in which sources and sinks were replenished three times over 24 hours (Fig. 4A), would permit high sink-to-source concentration differences without allowing equilibration of the reservoirs for the faster diffusing species. By contrast we reasoned that a single elevated dose of the macromolecule, BMP4, and small molecule, CHIR99021, would result in higher relative levels of CHIR99021 accumulation in the sink and depletion from the source given sufficient time for the BMP4 gradient to fully form. Thus, we used multiple dosings to help standardize our gradient formation protocol and compensate for differences in these molecules’ (and other signaling species that may be used in future applications) diffusion rates. Our established FEM framework predicted gradient generation over two phases of diffusion in pre-transfer pattern formation (Fig. 4B, left and center) and stability in the last stage, gradient transfer (Fig. 4B, right). The model showed multiple dosing resulted in the desired higher final source-end concentrations with minimal change to sink-end concentrations compared to single dosing (Fig. 4C).

Using this method to generate steep and stable morphogen gradients (Fig. 4A), we then investigated whether parallel long-range BMP4 and CHIR99021 gradients could direct hPSC differentiation patterning. We hypothesized that increasing concentrations of BMP4 and CHIR99021 along a gradient would correlate with increasing expression of early mesendoderm marker Brachyury (T), since BMP4 and Wnt signaling can support mesendoderm specification in a dose-dependent fashion^13,44^. To test this hypothesis, we used the initial steps of an hPSC differentiation protocol for mesoderm specification^45^ modified to include graded BMP4/CHIR99021 presentation for 24 hr (Fig. 4D “Transient Rock Inhibition”). We found that after exposure to BMP4/CHIR99021 gradients, hPSC cultures exhibited a gradient of T expression (Fig. 4E–E”), in agreement with our hypothesis.

Since previous studies to direct spatial patterning of hPSCs have triggered intrinsic self-organization by manipulating culture conditions such as cell density, geometric confinement, and signaling state^17–21,46^, we further sought to test whether BMP4/CHIR99021 gradients drive differential T expression in a manner that also depends on upstream culture methods. We varied the timing and duration of Rock inhibitor (Y-27632 Dihydrochloride) exposure upon initial cell plating and prior to gradient exposure (Fig. 4D), which we hypothesized would impact cell signaling status^47,48^ and survival^49^. We found that regardless of the upstream culture protocol, all cultures exhibited a patterned response in which T expression showed an increasing trend moving from the BMP4/CHIR99021 sink end of the gradient toward the source end (Fig. 4E–G”). These gradients in T expression were also evident when quantified relative to the Hoechst signal across the culture islands (Fig. 4H–J). Finer characteristics of T expression patterning depended on the upstream culture method, consistent with studies that showed intrinsic patterning arises from differential colony geometry, cell-cell junction formation, and cell distribution^18,19,21,50^. Cultures with shorter Rock inhibition exhibited a diffuse gradient of T expression, with foci containing central T-negative cells surrounded by cells positive for T expression (Fig. 4E–E” and H). Sustained rock inhibition resulted in a continuous monolayer with a gradient of amplified expression around the population’s periphery (Fig. 4F–F”). This treatment resulted in a more uniform relative signal gradient in the culture bulk and an increase in relative signal at the sink end boundary, though not to the same level as the source end (Fig. 4I). Finally, extended culture in stem cell maintenance medium provided cultures with more proliferation time and resulted in irregular patterns of T expression (Fig. 4G–G”) and a sharp increase in relative T signal at the culture X-direction midpoint (Fig. 4J). Thus, we observed visible and quantifiable mesoscale gradients in T expression across the cultures, but gradient patterns were not always evident at the higher resolution microscale (Fig. 4E’–G’), which demonstrated local cell-to-cell variability in response. Together, our data demonstrate that external morphogen gradients can be used to drive positional hPSC fate choice across hPSC cultures in a manner that integrates with the cell population’s initial culture status.

### User-defined human cell fate patterning

Although cells are believed to partition into regions with distinct fates in response to morphogen gradients, how these cell populations interpret morphogen gradient features such as amplitude, slope, and inflection remains an open question^4,7,51–53^. The ability to create tunable gradients that drive hPSC fate in a controlled *in vitro* environment could provide an important tool to address this question. We therefore next tested whether morphogen gradient features could be spatially tuned to directly control ordering of hPSC lineage decisions.

**Figure 5:**
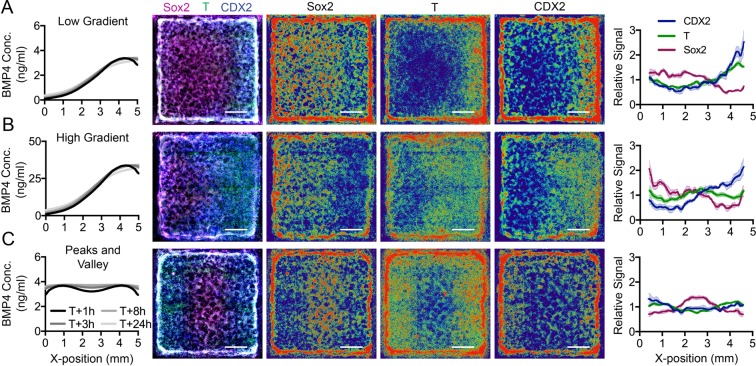
hPSC fate response to morphogen gradient tuning is lineage-dependent and driven by both morphogen level and gradient shape. We characterized Sox2, T, and CDX2 expression patterns represented as overlays of N = 4 replicates in multichannel composite (micrograph column 1) and single analyte heat maps (micrograph columns 2–4). Exposure of hPSCs to (**A**) low amplitude source, (**B**) high amplitude source, and (**C**) a peaks and valley-shaped gradients resulted in expression pattern changes in all three lineage markers (signals normalized to Hoechst and centered around their mean to give relative signal).

We assessed whether hPSCs respond differentially to gradients with different stimulus concentrations ranges (amplitudes) and profile shapes. We generated long-range BMP4/CHIR99021 gradients with varying dose ranges and inflection points and quantified hPSC fate patterning responses. Gradients generated from a low BMP concentration at the source (0.1–3.4 ng/ml concentration range predicted by FEM) resulted in hPSC differentiation profiles with decreasing Sox2 (pluripotency and differentiation toward ectoderm), increasing T (mesendoderm), and increasing CDX2 (trophectoderm/mesendoderm) expression toward the source end of the gradient (Fig. 5A). Gradients emanating from a high BMP4 concentration source (1–34 ng/ml by FEM; Fig. 5B) resulted in decreasing Sox2 expression, biphasic T expression (increasing then decreasing), and increasing CDX2 toward the source end. These patterns are consistent with established relationships between high levels of BMP4 and extraembryonic lineages (high CDX2 expression), and the presence of BMP4 at intermediate levels in regions undergoing mesendodermal specification (overlapping T and CDX2 expression)^54–56^. Finally, gradients with two equal and transient BMP4 maxima at either side of the island resulted in T and CDX2 expression patterns that mirrored the two maxima predicted at the beginning of pattern transfer to the culture and an inverted pattern of Sox2 expression (central maximum, Fig. 5C). Together, these data demonstrate that extrinsic, user-defined gradients with specified shape and amplitude can be used to differentially influence long-distance lineage patterning across hPSC cultures.

## Conclusions

We have developed a new method for creating soluble factor gradients *in vitro* and demonstrated its utility for patterning hPSC fate stratification. This approach enables offline generation of morphogen gradients with defined shape, amplitude, and dynamics followed by rapid pattern transfer to cells in two-dimensional culture. This technique leverages diffusive transfer, and in its current form, relies on transfer across a narrow (up to several hundred microns) gap between the transfer gel and cells. Future modifications to the device design or delivery method could enable dynamic delivery of multiple morphogens from sequentially applied devices or real-time transfer to cells as the gradients form *in situ*. Further, application of this concept together with 3D-printing or other biofabrication strategies could enable more complex geometric morphogen patterns or cellular (even 3D) topographies. This technology could provide a powerful complementary tool to probe how cells interpret specific gradient features (e.g., dose, slope, and inflection), filter signal noise, and interpret concentration dynamics in various phases of development, wound healing, and homeostasis^57–59^. It could also be useful in more translational applications, such as refining cell fate patterning for hPSC directed differentiation and construction of bioartificial engineered tissues.

## Methods

### Micromilling and polymer casting

Polystyrene devices and polystyrene molds for polydimethylsiloxane (PDMS) components were designed in SolidWorks v24 (Dassault Systèmes). SolidWorks parts were converted to G-code using SprutCAM 11 software (SPRUT Technology, Ltd.). Gel housing, gel molding bases, and molds for PDMS (Sylgard 184; Dow Corning, Cat# 1673921) were milled using a Tormach PCNC 770 mill (Tormach Inc.) as previously described^60^. Milled parts were fabricated from 2 mm- or 4 mm-thick polystyrene sheets (Goodfellow USA, Cat# 700-272-86). Single compartment OmniTray plates, surface treated for cell culture (ThermoFisher, Cat# 12-565-285) were customized by micromilling to accommodate patterned cell seeding and corresponding device placement. Briefly, 500 μm-deep reliefs were milled into the culture surface of plates leaving 12 square islands (5 mm × 5 mm) surrounded by rectangular troughs designed to receive gradient patterning devices. Gel housings for gradient devices were designed such that they included voids for agarose gel cups connected by a 500 μm-thick sheet of agarose molded across the bottom surface of the housing compartment. Housings also included outer walls that extended down 250 μm into the milled reliefs of OmniTrays. Feet positioned on the outside of the housing referenced first a PS mold that defined the bottom of the agarose sheet and cups (offset 100 µm above the feet bottom) and then the cell seeding surface of the plate (i.e. seeded island substrates). The feet were thereby used to precisely position the gel above cells on the substrate.

For PDMS casting, Sylgard 184 base and curing agent were mixed at a 10:1 ratio, degassed under vacuum for 20 min, and poured into molds. Molds were clamped against 4 mm-thick acrylic to seal in polymer. PDMS parts were then cured at 65 °C overnight, cooled, removed from molds, and cleared of any undesired polymer membranes. PDMS parts included seeding inserts that were placed into milled reliefs around culture islands creating a “well wall” around the perimeter that allowed for uniform seeding with a 30 μL volume. This volume created a uniform layer of seeding media without a meniscus. PDMS parts were also used to mold agarose cups in the reservoir voids of PS gel housings.

### Finite Element Modeling

We used COMSOL Multiphysics software (v5.1; COMSOL Inc.) to model gradient formation and transfer for soluble factors loaded in our source and sink fueled gradient devices. Estimated material properties and diffusion parameters for the diffusing species, and hydrogel media were obtained from the literature^37–40,61,62^. Transport through reservoir and cell media was modeled as free-diffusion.

### Cell culture

Human umbilical vein endothelial cells (HUVECs, Lonza, Cat# C2519A) were maintained in EGM-2 (Lonza, Cat# CC-3162) media and were passaged using 0.25% Trypsin EDTA (Corning, Cat# 25-052-CV). For CellTracker experiments, HUVECs were seeded at 20,000 cells/cm^2^. WTC-11 human induced pluripotent stem cells (hiPSCs, Coriell Institute, Cat# GM25256) were maintained in mTeSR 1 (STEMCELL Technologies, Cat# 85850) media on growth factor reduced Matrigel (Corning) coated culture plates and were passaged using StemPro Accutase (ThermoFisher, Cat# A1110501) in maintenance media containing Rock inhibitor Y-27632 (10 μM, R&D Systems, Cat# 1254). Media was changed 8–16 hr after seeding to maintenance media without Rock inhibitor. hiPSCs were seeded for experiments at 150,000 cells/cm^2^ on Matrigel (growth factor reduced, Corning, Cat# 356231) coated islands milled in OmniTrays. Culture prior to treatment with gradient devices was conducted using one of three protocols outlined in Fig. 4D. Base media, RPMI 1640 (Gibco, Cat# 11875119) with 1 × B27 Supplement minus insulin (ThermoFisher, Cat # A1895601) unless noted as mTeSR, contained supplements at the following concentrations: GSK-3 inhibitor CHIR99021 (CHIR, Tocris Bioscience, Cat# 4423), 1 μM; Rock inhibitor Y-27632, 10 μM; and recombinant Activin A (Activin, R&D Systems, Cat# 338-AC-050), 100 ng/mL.

### Gradient formation and transfer to cells

Agarose (SeaPlaque, Lonza, Cat# 50111) and housing composite gradient devices, which together allowed for defined diffusion patterns and transfer of the resultant gradients to cells, were fabricated as follows. We chose agarose as the matrix for gradient formation and transfer because it allowed for straightforward temperature-driven polymerization, and it had a combination of rigidity and porosity that allowed for robust micromolding and permitted the diffusion of both macro- and small molecules from their source reservoirs. We note that while other inert gels (e.g. poly-ethylene glycol or alginate) could also be compatible with this method, agarose is commonly used across various fields biological research and does not require chelating agents or chemical polymerization initiators necessary to polymerize other inert matrices. PS gel housings served as support structures and dictated the shape of agarose hydrogels. To define the base of the hydrogel, housings were placed on a PS molding base and ~250 µL of melted 3% agarose in phosphate buffered saline (PBS, ThermoFisher, Cat# 21600069) was added through the reservoir voids. The liquid agarose filled the space between the housing and bottom mold as well as the reservoir voids. For molding the reservoir cups, a PDMS top mold was comprised of two reservoir negatives (PDMS cubes) connected by a PDMS sheet which spanned and wrapped around the top surface of the housing device. The top mold was placed and pressed down with sterile forceps so that the reservoir negatives displaced excess agarose and defined the inner surfaces of the reservoir gel cups. Agarose was allowed to cool to room temperature and gel for 15–30 min, at which point the PDMS top molds were removed. To begin gradient formation the molded reservoir voids were filled with the appropriate source and sink media. Depending on the experiment, gradient formation via diffusion was allowed to proceed for 24 hrs with or without source and sink replenishment.

For FITC (Fluorescein 5-isothiocyanate, Sigma-Aldrich, Cat# 1245460250) and FITC-labled dextrans the formation of 1D gradients were monitored in devices with 5 mm separation between source and sink, a width of 5 mm, a connecting gel sheet thickness of 500 μm, and source and sink volumes of 30 μl. Diffusion from the source was visualized using FITC and FITC-conjugated 20 kDa (Sigma-Aldrich, Cat# 95648) and 70 kDa dextrans (Sigma-Aldrich, Cat# 54702) in 1× PBS. The gradient formation region was imaged over approximately 20 hours with source volume balanced with 1× PBS in the sink reservoir.

In CellTracker dye experiments, HUVECs were seeded on 5 mm × 5 mm square islands milled into cell culture treated omnitrays two days prior to pattern transfer. HUVECs were used to study gradient tuning because they readily form homogeneous and highly confluent cellular monolayers, which enabled quantification of fluorescence of single cells across the gradient transfer region. 24 hrs before pattern transfer, gradient formation was initiated by filling one reservoir in each device with CellTracker Red CMTPX Dye (ThermoFisher, Cat# C34552) and the other reservoir with CellTracker Green CMFDA Dye (ThermoFisher, Cat# C2925). CellTrackers were used at a 100 uM (10×) working concentration in 1× PBS. Immediately before pattern transfer to cells, source and sink reservoirs were emptied, the bottom surfaces of the gradient devices were rinsed with media. Devices were then placed in the relief surrounding each HUVEC-seeded island. Transfer of the gradient occurred over a 15 min incubation at RT. Following transfer, devices were removed, the media over each island was replaced, and the islands were live imaged.

For gradients intended for extended BMP4 (R&D Systems, Cat# 314-BP-010) and CHIR99021 exposure, the source and sink were replenished with new media periodically over 24 hrs at RT and before placement with 3 mg/ml collagen I solution containing the appropriate diffusing species. These devices were placed in a 5% CO_2_, 37 °C incubator for 60 min allowing the collagen to gel. Collagen gels stabilized the source and sink volumes through device removal from molding bases, rinsing of the bottom surface of the gel sheet, placement of the gradient device over cells, and extended incubation. Gradient devices were then removed from their bases, rinsed with media, and placed in island reliefs. The devices were moved to a 5% CO_2_, 37 °C incubator for 24 hr. Following treatment, devices were removed and cells were fixed with 4% paraformaldehyde (PFA, Electron Microscopy Sciences, Cat# 15714) for 10 min at RT in PBS for subsequent immunostaining.

### Immunohistochemistry

Following BMP4/CHIR99021 gradient exposure and fixation, WTC-11 hPSCs from a given experiment were (in parallel) permeabilized with 0.1% Triton X-100 (VWR, Cat# 97062-208) in PBS for 10 min at RT, and blocked with 1% bovine serum albumin (BSA; Sigma-Aldrich, Cat# A7906) in PBS containing 0.1% Tween-20 (PBST, VWR, Cat# 97062-332). Cells were incubated overnight at 4 °C with primary antibodies against Brachyury (raised in goat, 13 μg/mL; R&D Systems, Cat# AF2085), CDX2 (raised in rabbit, 1:90 dilution; Abcam, Cat# ab76541), and Sox2 (raised in mouse, 25 μg/mL; Novus Biologicals, Cat# MAB2018) in 1% BSA-PBST. Secondary antibodies (donkey anti-goat Alexa Fluor 488 conjugate, ThermoFisher, Cat# A-11055; donkey anti-rabbit Alexa Fluor 647 conjugate, ThermoFisher, Cat# A-31573; and donkey anti-mouse Alexa Fluor 594 conjugate, ThermoFisher, Cat# A-21203) were applied for 1 hr at RT with a Hoechst 33342 (10 μg/mL, ThermoFisher, Cat# H3570) counterstain. All staining steps were performed in parallel for a given experiment using the same stock and working solutions. Cells were rinsed in PBS and imaged using a Nikon Ti-E inverted microscope.

### Image analysis

FITC and FITC-labeled dextran gradient image capture and stitching was performed with a BD Pathway Bioimager and BD AttoVision v1.6/855 software (BD Biosciences, San Jose, CA). Gradient quantification was performed using timepoint-specific calibration image-based background corrections. Gradient region intensity profiles were tabulated in ImageJ. Edges of the stitched fields were normalized and intensities were baseline and maximum normalized so that relative fluorescence ranged from zero to one for each device. The average fluorescence intensity profiles for N = 3 replicates were fit with a 4^th^ order polynomial at each time point.

For quantification of CellTracker gradient transfer, image data was managed and processed using software called JEX, an open-source Java application for managing, databasing, and processing large amounts of data^63^. JEX was run through Eclipse v4.7 (Oxygen; The Eclipse Foundation). Multi-field images were stitched in the NIS-Elements (Nikon) software and background corrected in JEX using calibration images. Individual cells were identified as local maxima and intensities for all maxima were quantified in both fluorescence channel images. Data spreadsheets generated in JEX with ROI IDs, X-Y coordinates, and intensity measures were read by a MATLAB (MATLAB v8.1, MathWorks) script, which was used to plot log(signal ratio) versus X-position and to calculate the slope of the relationship (average of N = 6 ± SEM).

For morphogen gradient studies, background corrected (“Subtract Background” function in ImageJ with rolling ball radius = 200 pixels) immunostaining images were analyzed in MATLAB using a script that identified Hoechst positive (defined by user input) pixels and normalized the corresponding Brachyury, Sox2, and CDX2 signals for those pixels to their Hoechst signal intensity. Normalized signals were averaged (rolling average with a 50 pixel width) for each X-position and centered around the mean normalized signal across the island to obtain relative signals. To compare expression pattern changes in Brachyury expression or across the three lineage markers relative signals were plotted against X-position as averages of N = 3 or N = 4 replicates respectively with shading denoting SEM intervals.

### Statistical analysis

Statistical analysis was performed in GraphPad Prism v7.0c. Error bars denote standard error of the mean and statistical significance between multiple groups was assessed by ordinary one-way ANOVA with Tukey’s multiple comparisons post-hoc test. p < 0.05 was considered statistically significant.

## Supplementary information

Supplementary Information

## Data Availability

Data, device design files, and FEM modeling files generated during and/or analyzed during the current study are available from the corresponding author on reasonable request.
