# Supplementary material for: User-defined morphogen patterning for directing human cell fate stratification
Source: Sci Rep. 2019 Apr 23;9:6433. doi: 10.1038/s41598-019-42874-8 (PMC6478938; doi:10.1038/s41598-019-42874-8)
Supplement: Supplementary file 1 — Supplementary Information [file 41598_2019_42874_MOESM1_ESM.docx]

**Supplementary Information**

**User-defined morphogen patterning for directing human cell fate stratification**

Mary C. Regier^a,b,c,d^, Jacob J. Tokar^c,d^, Jay W. Warrick^c,d,e^, Lil Pabon^b,f^, Erwin Berthier^g^, David J. Beebe^c,d^, Kelly R. Stevens^a,b,f^*

^a^Department of Bioengineering, University of Washington, 98195 Seattle, USA

^b^Institute for Stem Cell and Regenerative Medicine, University of Washington, 98109 Seattle, USA

^c^Department of Biomedical Engineering, University of Wisconsin - Madison, 53706 Madison, USA

^d^Carbone Cancer Center, University of Wisconsin - Madison, 53792 Madison, USA

^e^McArdle Laboratory for Cancer Research, University of Wisconsin - Madison, 53705 Madison, USA

^f^Department of Pathology, University of Washington, 98195 Seattle, USA

^g^Department of Chemistry, University of Washington, 98195 Seattle, USA

*e-mail: ksteve@uw.edu

**
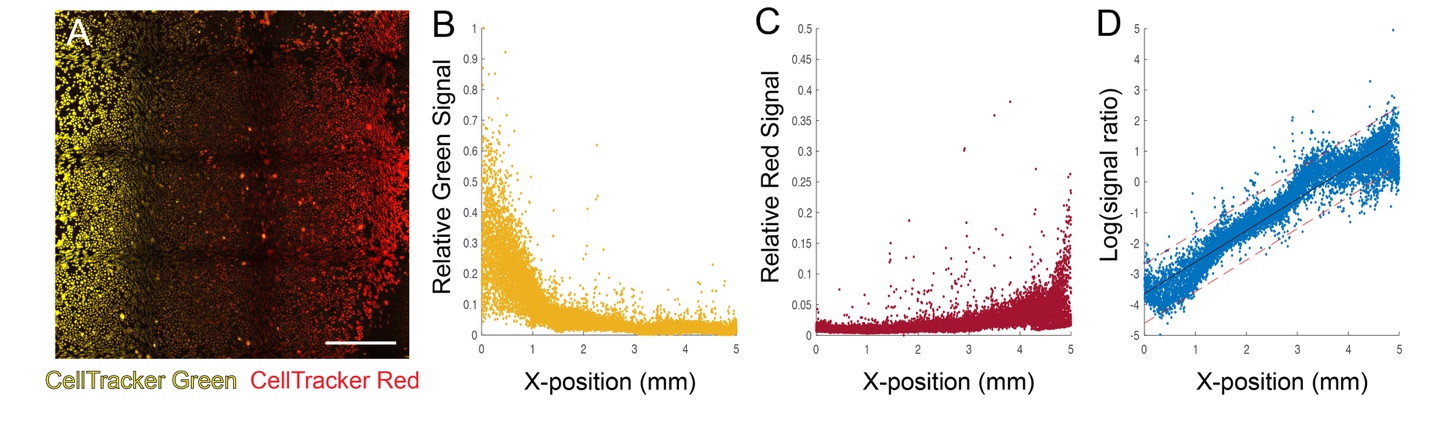
**

**Figure S1.** Signals patterned from opposing CellTracker gradients are position-dependent. (A) Transfer of opposing gradient of CellTrackers green and red patterned from a device with a 3mm reservoir separation to HUVECs resulted in opposing gradients of (B) green in-cell signals and (C) red in-cell signals. (D) The log() of the ratio of the two signals for each cell formed a linear trend when plotted against X-position (­­ – linear regression, – – 95% confidence intervals). Scale bar: (A) 1mm.
